# Ammonium-derived nitrous oxide is a global source in streams

**DOI:** 10.1038/s41467-024-48343-9

**Published:** 2024-05-14

**Authors:** Shanyun Wang, Bangrui Lan, Longbin Yu, Manyi Xiao, Liping Jiang, Yu Qin, Yucheng Jin, Yuting Zhou, Gawhar Armanbek, Jingchen Ma, Manting Wang, Mike S. M. Jetten, Hanqin Tian, Guibing Zhu, Yong-Guan Zhu

**Affiliations:** 1 Research Center for Eco-Environmental Sciences, Chinese Academy of Sciences, Beijing, 100085 China; 2 University of Chinese Academy of Sciences, Beijing, 100049 China; 3 Department of Microbiology, Radboud University Nijmegen, Nijmegen, AJ 6525 the Netherlands; 4 Center for Earth System Science and Global Sustainability, Schiller Institute for Integrated Science and Society, Boston College, Chestnut Hill, MA 02467 USA; 5 Department of Earth and Environmental Sciences, Boston College, Chestnut Hill, MA 02467 USA

**Keywords:** Freshwater ecology, Element cycles

## Abstract

Global riverine nitrous oxide (N_2_O) emissions have increased more than 4-fold in the last century. It has been estimated that the hyporheic zones in small streams alone may contribute approximately 85% of these N_2_O emissions. However, the mechanisms and pathways controlling hyporheic N_2_O production in stream ecosystems remain unknown. Here, we report that ammonia-derived pathways, rather than the nitrate-derived pathways, are the dominant hyporheic N_2_O sources (69.6 ± 2.1%) in agricultural streams around the world. The N_2_O fluxes are mainly in positive correlation with ammonia. The potential N_2_O metabolic pathways of metagenome-assembled genomes (MAGs) provides evidence that nitrifying bacteria contain greater abundances of N_2_O production-related genes than denitrifying bacteria. Taken together, this study highlights the importance of mitigating agriculturally derived ammonium in low-order agricultural streams in controlling N_2_O emissions. Global models of riverine ecosystems need to better represent ammonia-derived pathways for accurately estimating and predicting riverine N_2_O emissions.

## Abstract (web summary)

NH_4_^+^-derived pathways, rather than NO_3_^-^-derived pathway, are the dominant hyporheic N_2_O sources in lower-order streams. These findings provide insights into better estimation of N_2_O emissions in global models of riverine ecosystems and emphasize the importance of managing ammonium.

## Introduction

Nitrous oxide (N_2_O) is a potent greenhouse gas and dominant ozone-depleting substance, and its atmospheric mole fraction has increased by 23% since 1750^1^. Riverine N_2_O emissions have increased fourfold since 1900 and are important components of the global N_2_O budget^2^. A 2020 global modeling study^3^ showed that small streams (lower-order streams rather than high-order streams) contribute up to 85% of the global riverine nitrous oxide (N_2_O) emissions^3^, most of which are produced in hyporheic zones (beneath stream beds where stream waters exchange with adjacent sediments). However, in current process-based N_2_O models, the N_2_O/N_2_ ratio is used to represent the N_2_O production rate during denitrification^3^. Model parameters associated with microbially mediated hyporheic N_2_O production are poorly represented^3,4^. Although the IPCC Guidelines for national greenhouse gas inventories include N_2_O from nitrification^4^, previous studies have been focused on quantifying denitrification and N_2_O emissions resulting from nitrate (NO_3_^−^)^5,6^. Furthermore, it is difficult to distinguish between nitrifier denitrification, nitrification-coupled denitrification, and heterotrophic denitrification N_2_O production pathways, as these processes occur via homologous genes under similar conditions, such as low O_2_ conditions^7^. Hyporheic N_2_O production has been shown to be very heterogeneous^8–10^, but this heterogeneity is extremely difficult to account for in models and, therefore, is often excluded^5^. Currently, the microbial mechanisms underlying N_2_O production in hyporheic exchange zones are largely unknown. These processes may significantly influence global N_2_O budgets and be potentially severely underestimated.

Accordingly, the aim of this study was to investigate the microbial sources and mechanisms that are responsible for hyporheic N_2_O production in riverbed and riparian zone sediments. We first investigated the spatiotemporal characteristics of N_2_O production along transects of the Baiyangdian riverine network, the largest riverine network in the North China Plains, using isotopic ^15^N-^18^O and ^15^N tracing, quantitative reverse-transcription PCR (RT‒qPCR), and metagenome analysis at the site and regional scales. The North China Plains cover an area of 300,000 km^2^ and account for 23% of the Chinese cropland area. They account for more than 25% of global fertilizer N use, making them a global N_2_O emission hotspot^2,11,12^. However, N_2_O emissions from agricultural streams in China^13,14^ or in the North China Plains specifically^15,16^ have only been reported in a few studies. We further compared these results to those measured globally in temperate and tropical streams to obtain comprehensive conclusions.

## Results and discussion

### Site-scale investigation of heterogeneous N_2_O emission fluxes along a transect of a riverine hyporheic zone

**Fig. 1:**
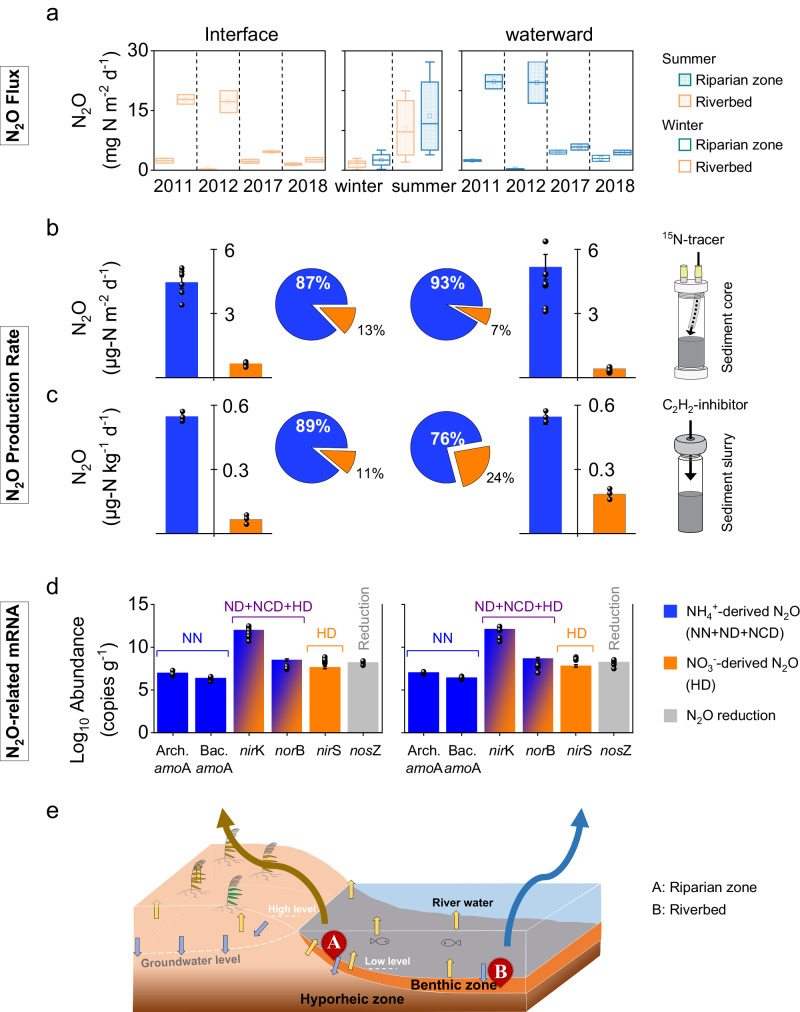
Spatial and temporal N_2_O emission fluxes and microbial production sources in the riparian zone and riverbed sediments along a transect of a riverine hyporheic zone at the site scale. **a** N_2_O emission flux over 4 years of monitoring (*n* = 48 independent experiments). For each box chart, the horizontal line indicates the median, the box represents the 25^th^ and 75^th^ percentiles, and the whisker shows the range from the 5^th^ to the 95^th^ percentile. **b**, **c** The microbial rate and contribution to N_2_O production via NH_4_^+^-derived and NO_3_^-^-derived processes based on semi-in situ sediment-core incubation via the ^15^N tracing method (**b**) and slurry incubation involving the C_2_H_2_-inhibitor method (**c**) (*n* = 6 and *n* = 3 biologically independent samples for core and slurry incubation, respectively). Data were presented as mean values ± SEM; **d** Transcript abundance of N_2_O-production related (*amo*A, *nor*B, *nir*S, and *nir*K) and N_2_O-reduction related (*nos*Z) genes (*n* = 3 biologically independent samples). Data were presented as mean values ± SEM. Here, *amo*A, ammonia monooxygenase gene encoded the NN pathway (NH_4_^+^ → NH_2_OH); *nir*SK, nitrite reductase genes (NO_2_^-^ → NO) encoded the ND (*nir*K only), NCD and HD pathways; *nor*B, nitric oxide reductase gene (NO → N_2_O) encoded the ND, NCD and HD pathways; and *nos*Z, nitrous oxide reductase gene (N_2_O → N_2_) encoded the NCD and HD pathways. **e** Schematic representation of the sampling sites along a transect of a riverine hyporheic zone.

Over a period of 4 years, the N_2_O emission fluxes in the riparian zone and riverbed sediments along a transect of a riverine hyporheic zone were continuously measured using a closed-chamber method (Fig. 1a and Supplementary Data S1). The emission fluxes were significantly greater in summer than in winter (*t*-test, *p* < 0.01), but there were no significant differences between the two zones. Ammonium (NH_4_^+^) showed the most positive correlation with N_2_O flux, irrespective of the sampling time and zone (Supplementary Data S2 and Table S1), as reported in many previous studies on rivers, agricultural catchments, and estuaries (Supplementary Table S2). In contrast, nitrate (NO_3_^-^) was negatively correlated with N_2_O flux, in contrast with previous findings^17,18^.

### Site-scale investigation of ^15^N tracing of the semi-in situ sediment core revealed NH_4_^+^-derived and NO_3_^−^-derived sources of hyporheic N_2_O production

Generally, N_2_O production is a biogeochemical process driven by microorganisms via two main microbial processes with four pathways: the NH_4_^+^-derived process (NH_4_^+^ as a substrate; includes nitrifier nitrification (NN), nitrifier denitrification (ND), and nitrification-coupled denitrification (NCD) pathways) and the NO_3_^−^-derived process (NO_3_^-^ as a substrate; heterotrophic denitrification (HD) pathway)^6,19–21^. Our results confirmed that the contribution of biotic N_2_O production in our samples was more than 92–95%, and abiotic processes accounted for less than 5–8% of the total N_2_O production (Supplementary Data S1, S2 and Fig. S1). A ^15^N tracing semi-in situ sediment-core incubation was performed to investigate the semi-in situ hyporheic N_2_O production rate and to clarify NH_4_^+^-derived and NO_3_^−^-derived sources without distinguishing the NH_4_^+^-derived process along the transect of the Xiaoqinghe River hyporheic zone (Fig. 1b and Supplementary Data S3). Interestingly, the NH_4_^+^-derived process was the dominant hyporheic N_2_O source in both the riparian zone (87.3 ± 3.9%) and the riverbed sediments (92.6 ± 5.6%), and the remaining N_2_O production could be attributed to the NO_3_^−^-derived process.

Furthermore, we used the 0.01% C_2_H_2_-inhibitor method to confirm the above results (Fig. 1c and Supplementary Data S1, S2). First, we found that the potential rate of biotic N_2_O production in the riparian zone (0.61 ± 0.03 μg N_2_O-N kg^−1^ h^−1^) was lower than that in the riverbed zone (0.73 ± 0.09 μg N_2_O-N kg^−1^ h^−1^), in agreement with the ^15^N semi-in situ sediment-core incubation results. However, this was not in agreement with the previous, which reported a higher N_2_O production rate in the riparian zone in different river systems^18,22,23^. In the riparian and riverbed zones, NH_4_^+^-derived N_2_O accounted for 89.2 ± 2.9% and 76.1 ± 2.6% of the total N_2_O produced, respectively. The NO_3_^−^-derived process resulted in the production of the remaining N_2_O. These findings differ from the prevailing opinion that NO_3_^−^-derived processes are the main contributors to N_2_O in riverine hyporheic zones^6,24^.

### Site-scale investigation of quantitative reverse-transcription PCR (RT‒qPCR) analysis of N_2_O-related gene mRNA

To further investigate the microbial mechanism and activity related to N_2_O production, the transcript abundances of microbial N_2_O-related genes were quantified via reverse-transcription PCR (RT‒qPCR) (Fig. 1d). The transcript abundances of all N_2_O-related (production and reduction) genes showed low heterogeneity between the riparian and riverbed zones. N_2_O production-related genes (*amo*A, *nor*B, *nir*S, and *nir*K) had greater transcriptional abundances than the reduction gene (*nos*Z) in both the riparian and riverbed zones, providing evidence that the hyporheic zone had greater potential for N_2_O production. RT‒qPCR cannot be used to distinguish both the *nir*K and *nor*B genes in the NH_4_^+^-derived and NO_3_^−^-derived N_2_O production pathways. However, we found that the transcript abundances of genes in the NH_4_^+^-derived N_2_O production pathway, including the *amo*A gene encoded the NN pathway and the *nor*B gene encoded the ND and NCD pathways, were greater than those of the *nor*B gene encoded the HD pathway, irrespective of sediment location (Fig. 1d).

### Regional-scale investigation of microbial pathways and key parameters influencing hyporheic N_2_O production

**Fig. 2:**
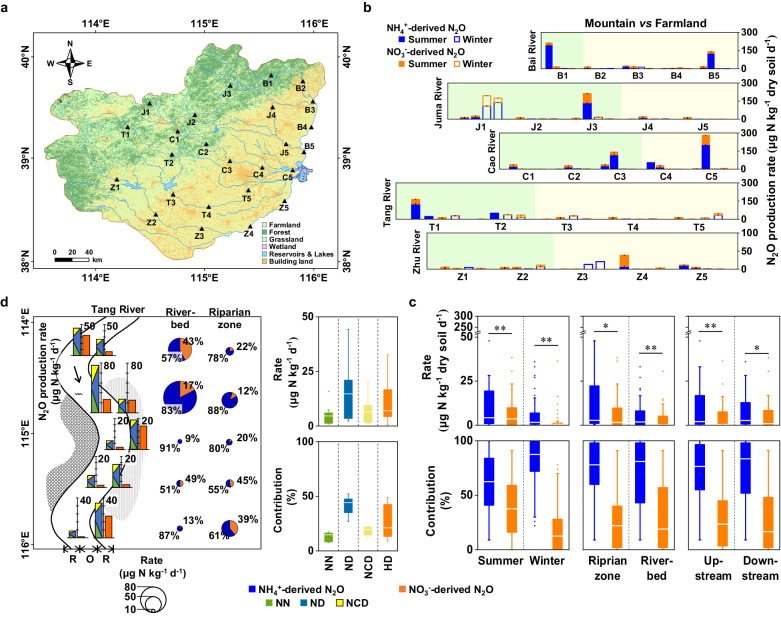
Biogeographical distribution of microbial N_2_O production sources in the riparian zone and riverbed sediments along transects of riverine hyporheic zones at the regional scale. **a** Overview of the Baiyangdian riverine network and the sampling sites; **b** Spatiotemporal distribution of the potential rate of NH_4_^+^-derived and NO_3_^-^-derived N_2_O production in the five streams (46–273 km length) of the Baiyangdian riverine network (*n* = 3 biologically independent samples). Data were presented as mean values ± SEM; **c** Spatiotemporal heterogeneity analyses of the potential rate and contribution of NH_4_^+^-derived and NO_3_^−^-derived pathways at the regional scale. *P* values were calculated with the two-tailed independent *t*-test (*p < 0.05, **p < 0.01 two-tailed; Except *n* = 44 and 56 independent experiments for upstream and downstream, respectively, *n* = 50 for other groups); **d** Potential rates and contributions of nitrifier nitrification (NN), nitrifier denitrification (ND), nitrification-coupled denitrification (NCD), and heterotrophic denitrification (HD) pathways in the sediments of the riverine hyporheic zones in the Tang River (*n* = 20 independent experiments). For each box chart, the horizontal line indicates the median, the box represents the 25th and 75th percentiles, and the whisker shows the range from the 5th to the 95th percentile. Bai River, B; Juma River, J; Cao River, C; Tang River, T; Zhulong River, Z; and Riparian zone, R; riverbed zone, O.

To support the above site-scale results, samples were also collected from 50 riparian zones and 50 riverbed sediments (0–20 cm depth) in 25 hyporheic zone transects at equal distances along five streams in the high- and low-water-level seasons (Fig. 2a, Supplementary Data S1, S2, and Fig. S2). The 0.01% C_2_H_2_ inhibitor results showed that N_2_O production in hyporheic sediments was dominated by NH_4_^+^-derived processes, the contribution of which to N_2_O production (72 ± 3%, *n* = 100) was significantly greater than that of NO_3_^−^-derived processes (28 ± 3%, *n* = 100) (paired *t*-test, *p* < 0.0001; Fig. 2b and Supplementary Data S2). There was less heterogeneity in hyporheic N_2_O emissions among the five different streams or in all streams at both the site and regional scales, but there was large temporal heterogeneity at the seasonal scale, with lower heterogeneity in winter (0.63 ± 0.22 mg N kg^−1^ soil d^−1^) than in summer (1.26 ± 0.34 mg N kg^−1^ soil d^−1^) (*p* < 0.05), and the values mostly depended on the NH_4_^+^ content (*r* = 0.851, *p* < 0.0001, *n* = 100) (Fig. 2b, c; Supplementary Fig. S2; Data S2; and Table S3). This result indicates that in hyporheic riverine sediments, microbial N_2_O generation is mainly driven by NH_4_^+^-derived processes rather than by NO_3_^−^-derived processes.

Furthermore, we used improved ^15^N-^18^O dual-isotope tracing to verify these results in one of the five streams, the Tang River. Based on our findings, NH_4_^+^-derived N_2_O production (73 ± 5%, *n* = 10) was significantly greater than NO_3_^-^-derived N_2_O production (27 ± 5%, *n* = 10) (paired *t*-test, *p* < 0.0001; Fig. 2d; Supplementary Fig. S2; and Data S2, S4), which was consistent with the above 0.01 % C_2_H_2_-inhibitor results. Among the NH_4_^+^-derived pathways, the ND pathway was dominant upstream (38 ± 4%, *n* = 4), downstream (44 ± 3%, *n* = 6), in riparian zone sediments (45 ± 3%, *n* = 5), and in riverbed sediments (38 ± 4%, *n* = 5), with little heterogeneity at both the site and regional scales. The remaining NH_4_^+^-derived N_2_O production was attributed to the NN and NCD pathways, with a range of 8–23%. These results showed that ND was an important pathway for N_2_O generation, which has long been overlooked but has recently been widely reported^25–29^. However, the biogeochemical mechanism underlying the role and key factors influencing this pathway are still unknown.

### Regional-scale investigation of potential metabolic N_2_O production mechanism

**Fig. 3:**
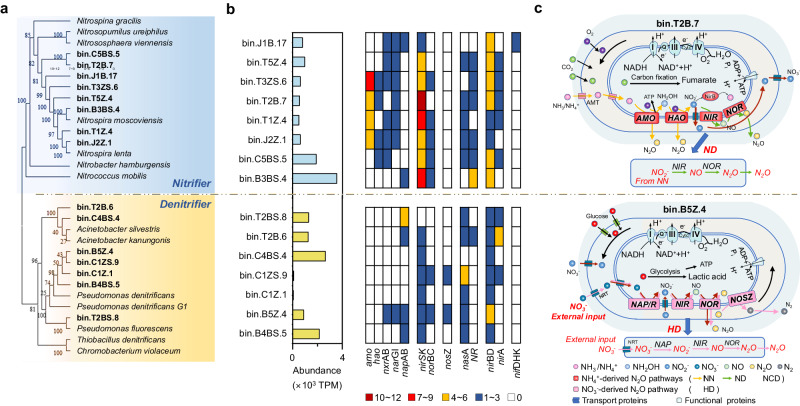
Metabolic N_2_O production mechanism. **a** Phylogenetic diagram of the genome associated with metagenomic recombinant binning. Based on the 16S rRNA sequence, phylogenetic relationships were calculated using the maximum likelihood method. The sequences associated with the N cycle based on the NCBI database and metagenome-assembled genomes (MAGs) identified in this study are shown in black and red, respectively. Each MAG was labeled as bin.“MAG source”.“MAG number” and included River (Bai River, B; Juma River, J; Cao River, C; Tang River, T and Zhulong River, Z), Site (1, 2, 3, 4, and 5), Zone (Riparian zone, R; Riverbed zone, O), and Season (Summer, S; Winter, W). The species, abundance, and metabolic path diagrams of nitrifiers and denitrifiers are shown above and below the orange dotted line. **b** Relative abundance of 15 MAGs and gene recombination and characterization of N_2_O production- and metabolism-related microbial processes. The cyan bar graph represents the relative abundance (TPM) of 15 MAGs. Different colors in the grid represent different gene abundances (TPMs). **c** Metabolic relationship of substances related to N_2_O production. The bin.T2B.7 genome and the bin.B5Z.4 genome represent nitrifiers and denitrifiers, respectively. The black arrows in the figure indicate material transfer, transformation, or electron transport. The yellow, green, brown, and pink arrows represent the NN, ND, NCD, and HD pathways, respectively, in the nitrogen cycle. Filled blocks of different shapes or colors represent different enzymes in cells.

As previously mentioned, the *nir* and *nor* genes are involved in the ND, NCD, and HD pathways; as a result, these processes cannot be distinguished based on the presence of these genes. To resolve this issue, species annotation was combined with functional gene annotation to study the genes related to nitrogen cycling in nitrifying and denitrifying bacteria and distinguish between the *nir* and *nor* genes in ND, NCD, and HD. The assembly and binning processes generated 198 high-quality (completeness >75%, contamination <15%) metagenome-assembled genomes (MAGs), 111 of which encoded genes involved in N_2_O production and reduction (Supplementary Data S5, S6). Through species and functional gene annotation, we obtained eight MAGs containing *amoABC*, *hao*, or *nxrAB* genes, which have been identified as nitrifying bacteria, and seven MAGs that contained the *nirK/S* gene, identified as denitrifying bacteria^30,31^. The annotation results of the species and functional genes were further verified via phylogenetic analysis (Fig. 3a). The relative abundances of nitrifying and denitrifying bacteria were 9524 and 8310 TPM, respectively (Fig. 3b).

Subsequently, the abundances of the N_2_O-producing genes *amo*ABC, *hao*, *nir*SK, and *nor* in nitrifying and denitrifying bacteria were determined (Fig. 3b and Supplementary Data S6). Traditionally, NH_4_^+^ oxidation is the rate-limiting step in nitrification^32–34^ and in the N cycle^35^. However, both the total and individual abundances of N_2_O-producing genes in nitrifying bacteria (26, 8, 48, and 6 TPM, respectively) were significantly greater than those in denitrifying bacteria (0, 0, 6, and 4 TPM, respectively). In contrast, the abundance of the N_2_O-reducing gene *nos*Z (N_2_O → N_2_) was 4 TPM in denitrifying bacteria, but it was not detected in nitrifying bacteria (Fig. 3b). These results further revealed the mechanism of microbial N_2_O production and highlighted the dominant role of nitrifying bacteria (NH_4_^+^-derived pathways) in N_2_O production.

The microbial mechanism of NH_4_^+^-derived and NO_3_^-^-derived N_2_O production was further revealed from the perspective of energy metabolism by metagenome binning analysis and is shown as a single-cell draft (Fig. 3c). The transmembrane transport of nitrite and nitrate, which are necessary substrates for ND and HD, respectively, is an energy-consuming process^36,37^. The NO_3_^−^ and NO_2_^−^ produced by nitrifying bacteria can be directly used in the ND pathway as substrates^38^, whereas denitrifying bacteria in the HD process need to consume adenosine triphosphate (ATP) to absorb NO_3_^−^ from the environment^36^. Additionally, the *nir*KS gene is significantly more abundant in nitrifying microorganisms than in denitrifying microorganisms (*p* = 0.003); hence, in the consumption of NO_2_^−^ for detoxification^39–41^, the ND pathway has greater potential to alleviate the toxic effects of nitrite on microorganisms^39–41^ than does the HD pathway. Furthermore, nitrifiers have the potential to produce large amounts of N_2_O because, based on reports to date, nitrifying bacteria do not contain N_2_O reductase^42^. Denitrifying bacteria have the *nos*Z gene encoding N_2_O reductase; hence, the N_2_O produced during NCD and HD can be further reduced to N_2_^43^. In contrast, there is no *nos*Z gene in nitrifying bacteria, and the N_2_O produced by NN and ND is not consumed within the same cells^7,27,44^ and can be released. Overall, there is greater potential for N_2_O production from the NH_4_^+^-derived pathways than from the NO_3_^−^-derived pathway because of energy savings, NO_2_^−^ detoxification, and the absence of N_2_O reductase.

### Global-scale investigation of hyporheic N_2_O production in streams

**Fig. 4:**
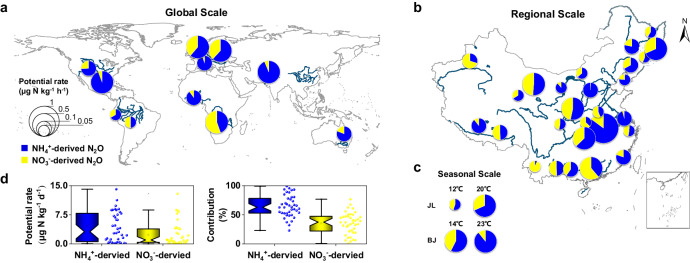
Significance and contribution of NH_4_^+^-derived pathways to riverine hyporheic N_2_O production at the global (a), regional (b), and temporal (c) scales and statistical analysis of rates and contributions (d). **a**–**c** The locations of the pie charts show the sampling sites; the sites for temporal-scale analysis are marked with solid orange circles. Detailed information on the sampling sites can be found in Supplementary Data S1. For each sampling site, the pie chart shows the total N_2_O production rate. The pie chart colors represent the relative contributions of the NH_4_^+^-derived (blue) and NO_3_^−^-derived (yellow) pathways to total N_2_O production. **d** Box charts (the horizontal line indicates the median, the box indicates the 25th and 75th percentiles, the whisker shows the range from the 5th to 95th percentiles, and the colors show the N_2_O production pathways) indicating the potential rate of N_2_O production via NH_4_^+^-derived and NO_3_^-^-derived pathways and their related contributions in riverine hyporheic zones worldwide (*n* = 41 independent experiments).

The above findings indicate that NH_4_^+^-derived pathways, rather than NO_3_^−^-derived pathways, largely influence hyporheic N_2_O production in the Yangtze, Yellow, Pearl, Yarlung Zangbo, Huai, Liao, Songhuajiang, and Heilongjiang river basins. To further expand our survey, we carried out a global spatiotemporal investigation across a wide range of streams around the world using the 0.01% C_2_H_2_ inhibitor method (Supplementary Data S1). Analysis conducted on a spatial scale showed that the rates of NH_4_^+^-derived N_2_O production were significantly greater than those of the NO_3_^−^-derived pathway at both the regional (4.56 ± 1.20 vs. 3.60 ± 1.68 μg N kg^−1^ d^−1^, *n* = 27) and global scales (6.00 ± 1.20 vs. 2.40 ± 0.72 μg N kg^−1^ d^−1^, *n* = 11, *p* = 0.003) (Fig. 4a, b and Supplementary Table S1). Seasonal scale analysis in the Buerhatong River (15–22 °C) and Xiaoqing River (14–23 °C) also revealed that the NH_4_^+^-derived N_2_O production rates (8.40 ± 2.40 μg N kg^−1^ d^−1^) were substantially greater than the NO_3_^-^-derived rates (3.60 ± 1.68 μg N kg^−1^ d^−1^; *n* = 4, *p* = 0.001) and were the main contributors to N_2_O production (67.5 ± 6.7%) (Fig. 4c). Statistical analysis at the spatial and temporal scales revealed a similar trend, namely, that NH_4_^+^-derived pathways contributed most significantly to N_2_O production (64.7 ± 2.9%) (Fig. 4d). In summary, NH_4_^+^-derived pathways are the dominant hyporheic N_2_O sources in low-order agricultural streams around the world.

Based on our results, the NH_4_^+^-derived process, rather than the NO_3_^−^-derived process, is the dominant hyporheic N_2_O source in lower-order agricultural streams. Together, these findings provide insights into better estimation of N_2_O emissions in global models of riverine ecosystems, which has been under debate for many years because of either under- or overestimation of riverine N_2_O budgets in IPCC assessments^2,5,39^. Furthermore, the results emphasize the importance of managing ammonium^11^. This is particularly important, as China has long been the largest ammonia fertilizer consumer in the world^12^. Approximately 60% of the fertilizer applied is not utilized by crops but leaches into stream systems, stimulating global climate warming; that is, the climate benefits of increased CO_2_ uptake via crop ammonia application are outweighed by stimulated greenhouse gas production from ammonia pollution^12^. Therefore, it is necessary to optimize fertilizer and ammonia management.

## Methods

### Study site and sampling

The Baiyangdian riverine network (113°40’–116°20’E, 38°10’–40°10’N) in the Haihe River Basin, with a total area of 3.12 × 10^4^ km^2^, is the largest riverine network in the North China Plains. The river network density is 0.50–0.99 km/km^2^, representing the upper level in China, with significant temporal distribution differences. Its upper and middle regions receive 30–40 first- or second-order tributaries or small streams with a total length of ~3000 km. These small streams drain various land areas in agricultural, urban, and mountain regions and are affected by various human activities and agricultural fertilization practices. Their catchment areas exceeded 5.5% of the agricultural area. First, a site-scale study was conducted on one of these small streams, the Xiaoqinghe River (Supplementary Data S1). Nine parallel in situ sediment cores (0–20 cm depth) were collected separately from the riparian (2 m away from the water‒soil transition zone) and riverbed (the middle of the river) zones. Six sediment cores were subjected to semi-in situ ^15^N-tracer assays, and the other three were subjected to 0.01% C_2_H_2_ inhibitor assays and molecular analysis. Furthermore, a regional-scale study was conducted on five rivers in the Baiyangdian riverine network (Fig. 2a and Supplementary Data S1). A total of 100 sediment samples were collected in the riparian zone and from riverbed sediments in the five rivers at five sampling sites along each river during the dry (Jan to Mar 2021) and rainy (Aug to Oct 2021) seasons. Then, we chose steams located adjacent to farmlands as the study sites, characterized by their land use type being cropland land^45^. Twenty-eight low-order agricultural streams were sampled in major river basins worldwide, including the Mississippi River, Colorado River (North America), Amazon River, Ucayali River (South America), Elbe River, Weser River, Po River (Europe), Niger River, Zambezi River (Africa), Murray River (Oceania), Indus River, Yangtze River, Yellow River, Pearl River, Yarlung Zangbo River, Huai River, Liao River, Songhuajiang River, and Heilongjiang River (Asia) (Supplementary Data S1).

All the sediment cores were collected in triplicate using an auger (Beijing New Landmark Soil Equipment, Beijing, China) with a plexiglass tube (5.5 cm diameter, 20 cm height). The site-scale sediment cores were stored in individual plexiglass tubes, with both nozzles of each plexiglass tube sealed with caps. The regional-scale and global-scale sediment cores were placed in individual sterile plastic bags. The collected sediment cores were immediately transported to a laboratory at 4 °C for subsequent analyses (Supplementary Data S1).

### ^15^N-tracer assay for semi-in situ sediment-core incubation

The potential rates of NH_4_^+^-derived and NO_3_^−^-derived N_2_O production were measured via the ^15^N-tracer semi-in situ incubation method for the sediment core. Once the cores were at the laboratory, the gas in the headspace of the plexiglass tube was evacuated and then adjusted to standard atmospheric pressure using high-purity Ar (99.99%; Beijing Huayuan Gas, Beijing, China). The oxygen content was immediately adjusted by the injection of a known volume of high-purity O_2_ (99.99%; Beijing Huayuan Gas) to the site oxygen content after the same volume of Ar was withdrawn from the headspace to balance the pressure. Two treatments for ^15^N enrichment were applied in triplicate: (i) ^15^NO_3_^−^ (^15^N at 99.19%) + ^14^NH_4_^+^ and (ii) ^15^NH_4_^+^ (^15^N at 99.16%) + ^14^NO_3_^−^. In each treatment, final enrichments of ^15^N-NH_4_^+^/NO_3_^−^ were added to 8.0 atom% ^15^N excess. The final N concentrations of both NH_4_^+^ and NO_3_^−^ remained close to the in situ concentrations in the sediments. The ^15^N-enriched cores were incubated under site temperature and oxygen conditions in a constant-temperature incubator. At defined intervals (0, 18, 36, 72, and 144 h), headspace gas was collected by using a 25-mL gas-tight syringe (Agilent, USA) and transferred into a 12-mL vacuum exetainer (Labco, UK). The ^15^NO_3_^−^ in the sediments was first converted by sponge cadmium (1.0 g) into ^15^NO_2_^−^ and then to ^29^N_2_. The ^15^NH_4_^+^ in the sediments was converted by hypobromite into ^30^N_2_.

### ^15^N-^18^O tracer assay for N_2_O production

A slightly modified version of a previously reported improved ^15^N-^18^O dual-isotope tracing method^7,19–21^ was used to carry out incubation experiments under site oxygen and temperature conditions. The improved ^15^N-^18^O dual tracing method was applied to 20 sediment samples from the Tanghe River, one of the five rivers in the Baiyangdian River. After visible roots and plant residues were removed, 5 g of homogeneous fresh sediment with 25 ml of overlying water close to the sediment (5:1, v/w) was placed into a 60-mL glass serum vial (Ochs Laborbedarf, Germany). Four treatments enriched in ^18^O and ^15^N were applied in triplicate: (i) H_2_^18^O (^18^O at 97.2%) + NH_4_^+^ + NO_3_^−^, (ii) N^18^O_3_^−^ (^18^O at 96.3%) + NH_4_^+^ + NO_3_^−^, (iii) ^15^NO_3_^−^ (^15^N at 99.19%) + NH_4_^+^ + NO_3_^−^, and (iv) ^15^NH_4_^+^ (^15^N at 99.16%) + NH_4_^+^ + NO_3_^−^. In all treatments, final enrichments of ^18^O-H_2_O/NO_3_^−^ and ^15^N-NH_4_^+^/NO_3_^−^ were added to 1.0 atom% ^18^O and 10 atom% ^15^N excess, respectively. The final N concentrations of both NH_4_^+^ and NO_3_^−^ were the site N concentrations in the fresh sediments. All vials were sealed with plugs (Ochs Laborbedarf, Germany) and aluminum crimp cap (Agilent, USA) septum-equipped lids during the incubation period. Subsequently, the gas in the vials was evacuated and then adjusted to standard atmospheric pressure using high-purity Ar (99.99%). Subsequently, the O_2_ concentration was immediately adjusted by the injection of a known volume of high-purity O_2_ (99.99%) to the site O_2_ concentration after the same volume of Ar was evacuated from the headspace to balance the pressure. The treatments were incubated at 60 rpm for 36 h to guarantee complete exposure of the sediments to all substrates under site temperatures. At the end of the incubation, the gas samples were transferred to 12-mL vacuum exetainers (Labco, UK) to quantify N_2_O concentrations.

### 0.01% C_2_H_2_ inhibitor assay

Three treatments were applied to the sediments at each scale in triplicate: (i) no treatment (control), (ii) treatment with 0.01% C_2_H_2_ (v/v; ammonia oxidation inhibitor), and (iii) treatment with ZnCl_2_ (600 μl, 7 M; biotic process inhibitor). The incubation conditions were the same as those in the ^15^N-^18^O experiment. The headspace gas was sampled with a locked syringe equipped with a Luer lock valve (25.0 ml; Agilent) and injected into a 12.0 ml vacuumed glass serum vial after 0, 3, 12, 24, and 36 h.

### N_2_O concentration, production rate, and contribution of ^15^N and ^18^O tracer assays

The N_2_O concentration was determined via a gas chromatograph (7890 A, Agilent, USA) with an autosampler (precisions ± 2.8%), while linear regression was carried out for the potential N_2_O production rate with incubation time, with coefficients of determination (R^2^) greater than 0.80.

The ^15^N and ^18^O signatures of N_2_O in gas samples were measured using an isotope-ratio mass spectrometer (IRMS and Precon, Delta V Advantage, Thermo Fisher Scientific, Bremen, Germany; precisions <0.04 ‰ δ^15^N and <0.07 ‰ δ^18^O, respectively). The relative contributions of microbial N_2_O production pathways (NH_4_^+^-derived pathways, NN, ND, and NCD; NO_3_^−^-derived pathway, HD) were calculated according to the methods of refs. ^20,21^.

### RNA extraction and quantitative reverse-transcription (RT‒qPCR) assays

In the site-scale study, RNA was extracted from three parallel sediment cores from both the riparian zone and riverbed sediments using an RNeasy Power Microbiome RNA Isolation Kit (QIAGEN, Hilden, Germany) according to the manufacturer’s protocol. RNA quality and concentration were estimated using a NanoDrop 2000 Spectrophotometer (NanoDrop Technologies, Wilmington, DE, USA).

Reverse transcription was performed with a PrimeScript^TM^ RT Reagent Kit with gDNA Eraser (Perfect Real Time) (TaKaRa, Dalian, China). All RT-qPCR analyses were performed on a sequence detection system (ABI 7500; Applied Biosystems, Foster City, CA, USA) with SYBR-Green fluorescent dye (TaKaRa). The copy numbers of N_2_O-related genes were quantified by using the specific primers for the archaeal *amo*A gene with Arch-amoAF and Arch-amoAR, the bacterial *amo*A gene with amoA-1F and amoA-2R, the denitrifier and ammonia oxidizer *nir*K gene with nirK-876F and nirK-1040R, the *nir*S gene with nirS-F and nirS-R, the denitrifier and ammonia oxidizer *nor*B gene with norB-F and norB-R, and the *nos*Z gene with nosZ-F and nosZ-R. All tests were conducted in triplicate with amplification efficiencies between 90% and 110% and correlation coefficients (*R*^2^) above 0.98. More details on the primers and thermal profiles are listed in Supplementary Table S4.

### DNA extraction, metagenomic library sequencing, N_2_O-related genome binning, taxonomic classification, functional annotation, and phylogenetic analyses

DNA was extracted from 100 regional-scale sediment cores by using the FastDNA Spin Kit for Soil (MP Biomedicals, Solon, OH, USA) according to the manufacturer’s protocol. DNA quality and concentration were estimated using a NanoDrop 2000 Spectrophotometer (NanoDrop Technologies). Approximately 1.5 μg of extracted DNA (per sample) was used for metagenomic library preparation and subsequent sequencing on the Illumina PE150 platform (150-bp paired-end) with a sequencing depth of 10 G. Clean data were generated from raw metagenomic reads after low-quality nucleotides and reads with any ambiguous base calls were filtered out using Kneaddata (github.com/biobakery/kneaddata), and the data were subsequently quality-checked using FastQC (Babraham Bioinformatics, Babraham Institute, Cambridge, UK) (Supplementary Data S5). The quality-controlled clean data were used to obtain contigs using Megahit^46^, while Bowtie2^47^ and Samtools^48^ were used for comparison and format conversion, respectively.

Metagenome binning was performed with contigs above 1500 bp using MetaWRAP (v 1.2.1)^49^. The obtained metagenome-assembled genomes (MAGs) were purified with RefineM^50^ to remove contaminating contigs (Supplementary Data S6). The MAGs were quality-checked by using CheckM^51^ and were dereplicated with dRep software^52^, and 198 MAGs with a degree of completion greater than 75% and a degree of contamination less than 15% were selected for subsequent analysis^52^. The Quant_bins module in MetaWRAP (salmon algorithm)^53^ was used to calculate the average relative abundance. The taxonomic affiliation of the MAGs was determined by GTDB-Tk v2.3.0^54^. Functional gene and protein annotation for the 198 MAGs was performed against the KEGG, NCyc^55^, COG, and GO databases at an e value <1e^−5^. According to taxonomy affiliation and functional gene and protein annotation, the MAGs containing *amo*, *hao*, or *nxr*AB genes and those containing the *nir*KS^56,57^ gene were identified to belong to nitrifying and denitrifying bacteria, respectively. A phylogenetic tree of high-quality MAGs was generated by the maximum likelihood statistical method with the nearest-neighbor-interchange (NNI) ML heuristic method in MEGA 11 software^58^.

### Analysis of environmental variables

The sediment NH_4_^+^, NO_3_^−^, NO_2_^−^, TOM, TN, and TP concentrations were determined^59,60^. The oxygen concentration and temperature in sediments were measured in situ by using a Pocket Oxygen Meter (FireStingGO2, PyroScience GmbH, Germany). The details are shown in the Supplementary Information.

### Statistical analysis

The mean values, *t*-tests, Spearman’s correlations, and linear regression analyses were performed by using Statistical Product and Service Solutions 18.0 software (SPSS Inc., USA). The significance level was to α = 0.05 (*p* value ≤0.05).

### Reporting summary

Further information on research design is available in the Nature Portfolio Reporting Summary linked to this article.

### Supplementary information

Supplementary Information

Peer Review File

Description of Additional Supplementary Files

Supplementary Data 1

Supplementary Data 2

Supplementary Data 3

Supplementary Data 4

Supplementary Data 5

Supplementary Data 6

Reporting Summary

## Data Availability

The data generated in this study are provided within the article, Supplementary Information, and Supplementary Data files. Metagenomic sequencing data and metagenome-assembled genomes are available in the NCBI Sequence Read Archive (SRA) under the accession codes PRJNA943572 and PRJNA1031250, respectively.
