# Author Correction: Ammonium-derived nitrous oxide is a global source in streams

**DOI:** 10.1038/s41467-024-49020-7

**Published:** 2024-05-28

**Authors:** Shanyun Wang, Bangrui Lan, Longbin Yu, Manyi Xiao, Liping Jiang, Yu Qin, Yucheng Jin, Yuting Zhou, Gawhar Armanbek, Jingchen Ma, Manting Wang, Mike S. M. Jetten, Hanqin Tian, Guibing Zhu, Yong-Guan Zhu

**Affiliations:** 1 Research Center for Eco-Environmental Sciences, Chinese Academy of Sciences, Beijing, 100085 China; 2 University of Chinese Academy of Sciences, Beijing, 100049 China; 3 Department of Microbiology, Radboud University Nijmegen, Nijmegen, AJ 6525 the Netherlands; 4 Center for Earth System Science and Global Sustainability, Schiller Institute for Integrated Science and Society, Boston College, Chestnut Hill, MA 02467 USA; 5 Department of Earth and Environmental Sciences, Boston College, Chestnut Hill, MA 02467 USA

**Keywords:** Freshwater ecology, Element cycles

Correction to: *Nature Communications* 10.1038/s41467-024-48343-9, published online 14 May 2024

In this article, the author name Hanqin Tian was incorrectly written as Hangin Tian. The original article has been corrected.

