# Supplementary material for: Ammonium-derived nitrous oxide is a global source in streams
Source: Nat Commun. 2024 May 14;15:4085. doi: 10.1038/s41467-024-48343-9 (PMC11094135; doi:10.1038/s41467-024-48343-9)
Supplement: Supplementary file 1 — Supplementary Information [file 41467_2024_48343_MOESM1_ESM.pdf]

# Supplementary Information

## Ammonium-derived nitrous oxide is a global source in streams

Shanyun Wang[1,2], Bangrui Lan[1,2], Longbin Yu[1,2], Manyi Xiao[1], Liping Jiang[1,2], Yu Qin[1,2], Yucheng Jin[1], Yuting Zhou[1], Gawhar Armanbek[1,2], Jingchen Ma[1], Manting Wang[1], Mike S. M. Jetten[3], Hangin Tian[4,5], Guibing Zhu[1,2*], Yong-Guan Zhu[1,2]

1. Research Center for Eco-Environmental Sciences, Chinese Academy of Sciences, Beijing, 100085, China.
2. University of Chinese Academy of Sciences, Beijing 100049, China.
3. Department of Microbiology, Radboud University Nijmegen, the Netherlands.
4. Center for Earth System Science and Global Sustainability, Schiller Institute for Integrated Science and Society, Boston College, Chestnut Hill, MA 02467, USA.
5. Department of Earth and Environmental Sciences, Boston College, Chestnut Hill, MA 02467, USA.

*Corresponding author

Prof. Dr. Guibing Zhu

Key Laboratory of Drinking Water Science and Technology, Research Center for Eco-Environmental Sciences, Chinese Academy of Sciences, Beijing 100085, China

E-mail: gbzhu@rcees.ac.cn

# Summary

Here, we provide supplementary material such as methodologies, complementary data, and experimental analyses. We also provide supplementary figures and tables to illustrate the main text.

# Supplementary methods

## $N_2O$ concentration measuring and fluxes

The closed-chamber technique was applied to measure $N_2O$ emission flux in riparian and open water zones throughout a period of 4 years (2011, 2012, 2017, and 2018). The stainless-steel chambers consisted of two parts: pedestal and upper chamber. The pedestal was 25 cm high, with an internal diameter of 40 cm. The lower rim was sharpened to be driven into the sediment or positioned below the surface of the water using a buoyancy device to keep it afloat, and the upper rim had a 2 × 2 cm gutter around the outside that could be filled with water to make an airtight seal within the upper chambers. The upper chamber (h = 45 cm) was equipped with two battery-driven brushless fans, for mixing of the chamber headspace, and one temperature probe.

The $N_2O$ concentrations were measured shortly after sampling by gas chromatography (Agilent 4890D) with an electron capture detector (ECD). The temperature of the ECD was 330°C, while that of the oven was 55°C. The precision of the $N_2O$ analyses was ±2.8 %, based on replicate analysis of standard gas. When the temperature was 25°C and the air pressure was 1,000 hPa, the minimum detectable fluxes were 28, 14, and 9 mg $m^{-2}$ $h^{-1}$ in gas sampling intervals of 5, 10, and 15 min, respectively. A standard gas was analyzed after every six samples. The $N_2O$ flux was calculated from the linear change of its concentration in the chamber headspace as a function of time, base area, chamber volume, and molar volume of $N_2O$ at chamber headspace air temperature. The coefficient of determination ($R^2$) of the linear regression were greater than 0.60 for most data sets.

## Analytical procedures of environmental variables

Sediment $NH_4^+$, $NO_2^-$, and $NO_3^-$ concentrations were measured using a SEAL Auto-Analyzer 3 HR (Seal Analytical, UK) after extraction with 2 M KCl (1:5 wt/vol). The detection limits were 0.015, 0.015, and 0.03 mg $kg^{-1}$, respectively. Total nitrogen (TN), total carbon (TC), and total sulfur (TS) were determined using a VarioEL III Analyzer (Elementar Analysen System GmbH, Germany), with detection limits of 0.05, 0.2, and 0.25 mg $kg^{-1}$, respectively. The pH of the sediment was determined using a DELTA 320 pH Analyzer (Mettler Toledo, USA) after shaking a dry sediment : water (1:5 wt/vol) suspension for 30 min. Triplicates were run for QA/QC.

## DNA extraction, Metagenomic library sequencing, $N_2O$-related genome binning, taxonomic classification, and functional annotation

DNA was extracted from 100 regional-scale sediment cores by using the FastDNA Spin Kit for Soil (MP Biomedicals, Solon, OH, USA) according to the manufacturer's protocol. DNA quality and concentration were estimated using the NanoDrop 2000 Spectrophotometer (NanoDrop Technologies, Wilmington, DE, USA). Approximately 1.5 μg of extracted DNA (per sample) was used for metagenomic library preparation and subsequent sequencing on the Illumina PE150 (150-bp paired-end) with the sequencing depth of 10G. Quality control and host filtering were carried out on the original metagenomic data to obtain clean data using Kneaddata (github.com/biobakery/kneaddata), and subsequently quality-checked using FastQC (Babraham Bioinformatics, Babraham institute, Cambridge, UK) (Supplementary Data S5). After quality control, we assembled the clean reads sequencing data to obtain contigs using Megahit[1], while Bowtie2[2] and Samtools[3] were used for comparison and format conversion, respectively.

After obtaining the contig depth data, metagenome binning was performed using MetaWRAP (version 1.2.1)[4], including CONCOCT[5], MaxBin2[6], and Metabat2[7] (contigs above 1,500 bp for binning). The obtained metagenome-assembled genomes (MAGs) were purified with RefineM[8] to remove the contaminating contigs (Supplementary Data S6). We used CheckM[9] to perform a quality check on each MAG to obtain the completion and contamination information of the original MAGs. The MAGs were dereplicated with the dRep software[10], and MAGs with a completion degree greater than 75% and a pollution degree of less than 15% (a total of 198) were selected for subsequent analysis[10]. The Quant_bins module in MetaWRAP (salmon algorithm)[11] was used to calculate the average bin abundance. Taxonomy affiliation of MAGs was determined by GTDB-Tk v2.3.0[12]. Functional gene and protein annotation for 198 MAGs was performed against KEGG, NCyc[13], COG, and GO databases. The e-value cutoff in the blast of MAGs is $1e^{-5}$. According to the results of taxonomy affiliation and functional gene and protein annotation, the MAGs containing *amo*, *hao*, or *nxr*A/B genes and those containing the *nir*K gene[14,15] were identified as nitrifying and denitrifying bacteria, respectively, and were selected as subjects of our research.

## Phylogenetic tree of MAGs

Phylogenetic analysis of the metagenome-assembled genomes (MAGs) associated with metagenomic recombinant binning was completed by MEGA 11 software[16,17]. The reference species were 16S rRNA sequences associated with N-cycle from NCBI database. The 16S rRNA sequences of the 15 MAGs were extracted by “barrnap”[18]. The sequences of reference species and MAGs were combined and aligned using ClustalW method with MEGA 11 software. The aligned sequences were used for phylogenetic analysis. The phylogenetic tree was constructed using maximum likelihood method with MEGA 11 software.

## Relative abundance, functional gene, and metabolic pathways of MAGs

For each MAG, the bar charts represent the relative abundances and the grid charts represent the numbers of N-cycle functional gene, which were computed from the assembly and binning processes of metagenome. The annotated nitrifiers and denitrifiers were further reorganized to harbor four $N_2O$-production pathways, including nitrifier nitrification (NN), nitrifier denitrification (ND), nitrification-coupled denitrification (NCD) in nitrifiers and heterotrophic denitrification (HD) in denitrifiers. Functional genes *amo*, *hao* and *nxr* were the indicator of nitrification process, and genes *nap*A/B, *nir*S/K, *nor*B/C and *nos*Z would be believed to support denitrification process[19]. The bar chart of relative abundance and the grid chart of N-cycle gene numbers were conducted by Origin 2021 software. The metabolic pathway map was draw refer to Daims et al. (2015)[19] and Nunzia et al. (2021)[20] by Microsoft PowerPoint.

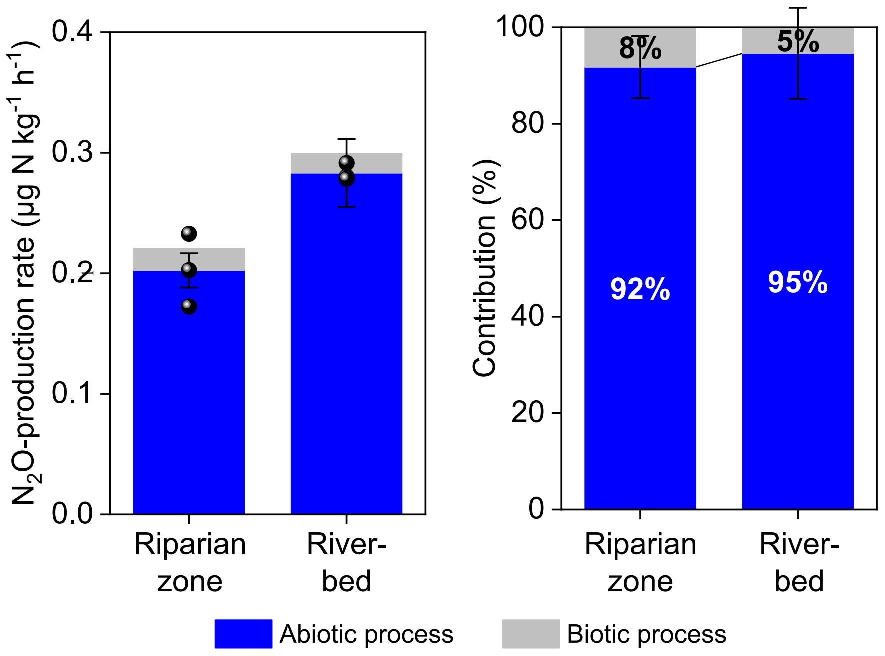


Supplementary Figure S1 The potential rates and roles of abiotic and biotic processes on $N_2O$ production in the riparian zone and open water sediments at site-scale investigation (n=3 biologically independent samples). Data are presented as mean values +/- SEM.

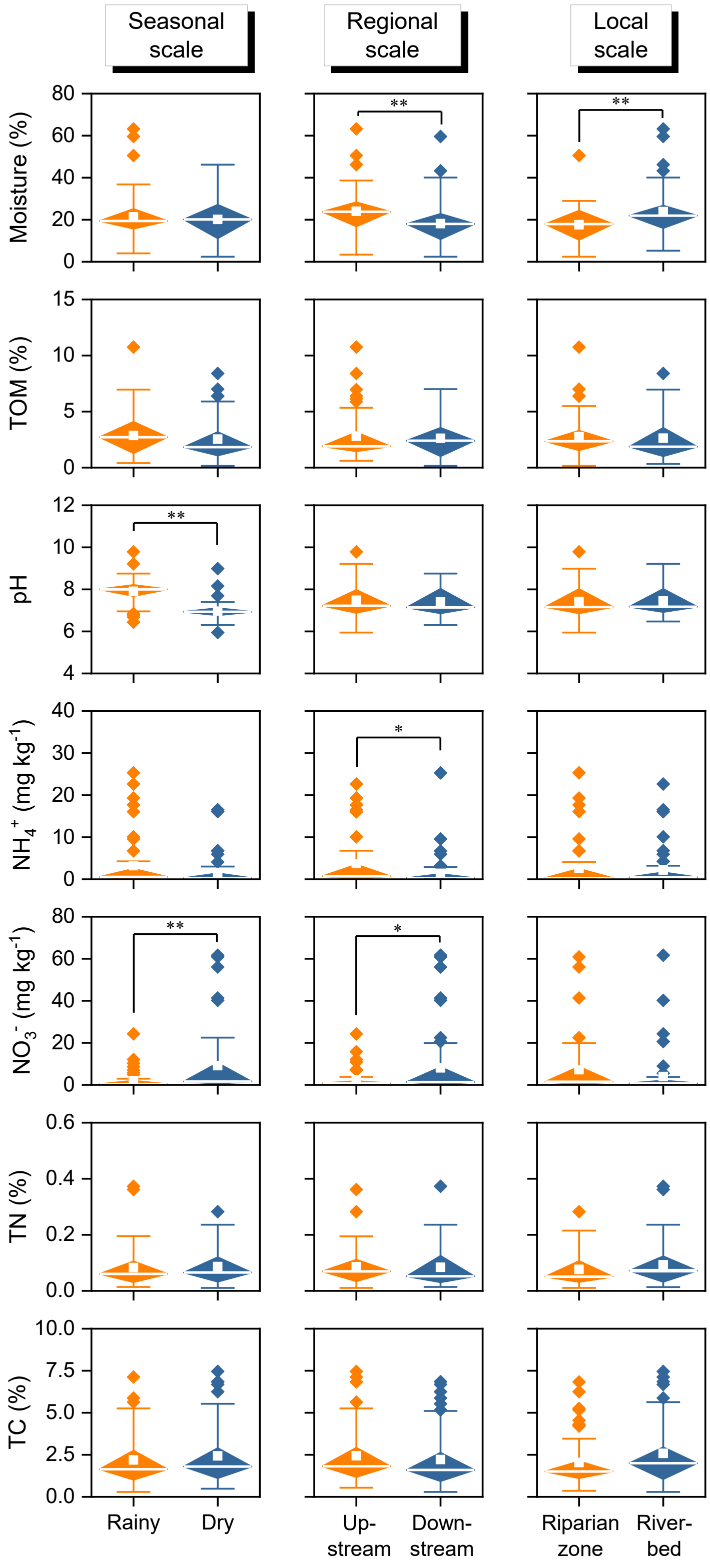


Supplementary Figure S2 The physicochemical parameters of five agricultural streams in the Baiyangdian Basin. P values were calculated with the two-tailed Independent t-Test (* $p < 0.05$, ** $p < 0.01$ 2-tailed; Except n=44 and 56 independent experiments for upstream and downstreams, respectively, n=50 for other groups)

Supplementary Table S1 Spearman correlation analysis between the $N_2O$ emission flux and physico-chemical characteristics in site-scale investigation.

| | | | $NH_4^+$ | $NO_x^-$ | TN | TP | pH | TOM | MC | DO in sediment |
|---|---|---|---|---|---|---|---|---|---|---|
| *$N_2O$ emission flux* | | | | | | | | | | |
| All Data | | *p* | 0.385 | -0.362 | 0.112 | -0.401 | 0.001 | 0.143 | -0.048 | -0.405 |
| | | $R^2$ | 0.141 | 0.168 | 0.728 | 0.325 | 0.996 | 0.598 | 0.911 | 0.32 |
| Yearly | 2011 | *p* | 0.8 | -0.8 | 0.8 | . | -1.000** | 0.4 | 0.8 | . |
| | | $R^2$ | 0.2 | 0.2 | 0.2 | . | . | 0.6 | 0.2 | . |
| | 2012 | *p* | 0.8 | -0.6 | . | . | 0.8 | 0.2 | -0.6 | . |
| | | $R^2$ | 0.2 | 0.4 | . | . | 0.2 | 0.8 | 0.4 | . |
| | 2017 | *p* | 0.4 | 0.105 | -0.2 | -0.6 | -0.4 | -0.4 | . | 0 |
| | | $R^2$ | 0.6 | 0.895 | 0.8 | 0.4 | 0.6 | 0.6 | . | 1 |
| | 2018 | *p* | 1.000** | -0.6 | -0.949 | 0 | 0.4 | 0.316 | . | -0.4 |
| | | $R^2$ | . | 0.4 | 0.051 | 1 | 0.6 | 0.684 | . | 0.6 |
| Seasonal | summer | *p* | 0.048 | -0.69 | 0.6 | -0.8 | -0.762* | -0.024 | 0.8 | 0.2 |
| | | $R^2$ | 0.911 | 0.058 | 0.208 | 0.2 | 0.028 | 0.955 | 0.2 | 0.8 |
| | winter | *p* | 0.619 | 0.357 | -0.086 | 0.894 | 0.807* | 0.381 | -0.6 | -0.8 |
| | | $R^2$ | 0.102 | 0.385 | 0.872 | 0.106 | 0.015 | 0.352 | 0.4 | 0.2 |
| Site-scale | Riparian | *p* | 0.143 | -0.238 | 0.371 | -0.316 | 0.395 | 0.048 | -0.4 | -0.4 |
| | | $R^2$ | 0.736 | 0.57 | 0.468 | 0.684 | 0.333 | 0.911 | 0.6 | 0.6 |
| | Openwater | *p* | 0.31 | -0.214 | -0.116 | -0.632 | -0.156 | 0.238 | -0.2 | -0.8 |
| | | $R^2$ | 0.456 | 0.61 | 0.827 | 0.368 | 0.713 | 0.57 | 0.8 | 0.2 |

Supplementary Table S2 Brief summary of $N_2O$ flux in the riparian zone and open water sediment.

| Site | Sampling Location | $NH_4^+$ (mg N L$^{-1}$) | $N_2O$ Flux (μg · N m$^{-2}$ · h$^{-1}$) | Temperature ($^{O}$C) | Ref. |
|---|---|---|---|---|---|
| The Yangtze Delta plain river net, Shanghai City | 30°27′00″–31°51′00″N 121°10′00″–121°45′00″E | 0.014 to 1.16 (mean 0.40±0.31) | 61.32±24.92 to 18284±3752 (mean 6608±1780.8) (Open water) | 28.9±1.27 (July) | 22 |
| Fengle River<br>Hangbu River<br>Nanfei River<br>(Chaohu Basin) | 30°58′00″–32°58′00″N, 116°24′30″–118°00′00″E | Mean: 0.28<br>Mean: 0.27<br>Mean: 12.26 | 10.01 to 88.77<br>7.56 to 18.32<br>10.91 to 947.69<br>(Open water) | 6.5–30.0<br>7.0–31.0<br>7.0–30.5<br>(Jun to Dec) | 23 |
| Nanfei River[a], Chaohu Basin | 31°49′N, 117°22′E | Mean: 12.54 | 1.53 to 2453 (mean 529) (Open water) | 7.0-30.5 (monthly in 2006 and 2009) | 24 |
| Chaohu lake | 31°25″28″–31°43′28″N, 117°16′54″–117°51′46″E | 0.35±0.16 | 24.1±29.8 (Open water) | Mean 18.9 (January, June, October, and August) | 25 |
| Poyang Lake Nanjishan wetland | 28°52′21″–29°06′46″N, 116°10′24″–116°23′50″E | 0.03 to 0.30 | −9.73 to 127 (Open water) | 28.3–31.4 (15 July 2013 to 10 August) | 26 |
| Jiaodu River<br>Shatang River<br>Dapu River<br>(Estuary of Taihu Lake) | 30°05′–32°08′N, 119°08′–121°55′E | 1.50±0.60<br>0.52±0.37<br>0.52±0.70 | 53.04<br>41.34<br>19.31<br>(Open water) | 11.0±0.25<br>12.1±0.25<br>12.1±0.10<br>(November) | 27 |
| Dongtan saltmarsh wetland, The Yangtze estuary | 30°25′–31°38′N, 121°50′–122°05′E | 14.96±0.40 – 13.11±0.47 mg kg$^{-1}$ soil | 10.64-21; 3.64-4.48 ng g$^{-1}$ soil h$^{-1}$ (Riparian) | 32 and 5 (July and January) | 28 |
| The Yangtze Estuary | 30°50′–31°50′N, 120°30′–122°00′E | 24.46±8.15 to 146.97±7.68 mg kg$^{-1}$ soil | 88.48±2.52 to 401.52±109.76 (Riparian) | 32 and 5 (January and July) | 29 |
| Bramble Bay<br>Deception Bay | 27°28′00″–27°46′00″S, 153°02′00″–153°03′00″E | | 11.21 to 73.59<br>12.12 to 82.97<br>(Open water) | 17.8–26.8<br>17.0–29.4<br>(October 2010 to August 2012) | 30 |
| Riverine wetlands in Columbus, Ohio, USA | 39°59′N, 82°59′W | | -1.51 to 91.64<br>-8.00 to 26.07<br>(High marsh<br>Low marsh) | 1.34-26.62<br>1.44-27.15<br>(June 2003 to December 2005) | 31 |
| Nenjiang Basin, China | 45°53′58″–45° 57′59″N, 123°57′35″–123°59′55″E | | -0.77±1.24 (Open water) | -16.7-23.7 (2009-2010) | 32 |
| Min River Basin of Fujian Province | 24°36′39″–25°00′56″N, 117°00′24″–117°69′50″E | 0.16–1.68<br>0.11–1.82<br>0.01–0.67 | 0.30 to 4.40<br>−1.53 to 5.62<br>−2.10 to 3.81<br>(Open water) | 28.1–34.1<br>19.8–32.1<br>21.3–34.1<br>(July 2021) | 33 |
| Streams, Sweden agricultural catchment | 59°85′N, 17°64′E | | -5.7 to 3449.7<br>Mean 108.2 | 0.81-19.17 (Dec. 2014–Aug. 2015.) | 34 |
| Xin'an jiang Reservoir, China | 29°28′–29°58′N, 118°42′–118°59′E | | 50.05 to 256.72 (Reservoir) | Mean 17.7 (Dec 2014 and Dec 2015) | 35 |

a. The Nanfei River is an urban river (urban effluents >70%)

Supplementary Table S3 Spearman correlation analysis between $N_2O$ production rates and physico-chemical characteristics in regional-scale investigation.

| | | Total Production Rate | Abiotic Process | Biotic Process | Ammonia oxidation | Heterotrophic Denitrification | Contribution of AO | Contribution of HD |
|---|---|---|---|---|---|---|---|---|
| MC | *p* | 0.301** | 0.151 | 0.280** | 0.284** | 0.062 | 0.07 | -0.069 |
| | $R^2$ | 0.002 | 0.134 | 0.005 | 0.004 | 0.541 | 0.487 | 0.498 |
| TOM | *p* | 0.177 | 0.051 | 0.178 | 0.188 | 0.084 | 0.114 | -0.12 |
| | $R^2$ | 0.078 | 0.612 | 0.076 | 0.061 | 0.406 | 0.259 | 0.235 |
| pH | *p* | -0.027 | -0.314** | 0.017 | -0.052 | 0.245* | -0.360** | 0.361** |
| | $R^2$ | 0.793 | 0.001 | 0.865 | 0.61 | 0.014 | 0 | 0 |
| $NH_4^+$ | *p* | 0.851** | 0.178 | 0.908** | 0.945** | 0.535** | 0.064 | -0.065 |
| | $R^2$ | 0 | 0.077 | 0 | 0 | 0 | 0.525 | 0.519 |
| $NO_3^-$ | *p* | -0.027 | 0.113 | -0.044 | -0.007 | -0.035 | 0.173 | -0.178 |
| | $R^2$ | 0.786 | 0.264 | 0.663 | 0.947 | 0.73 | 0.085 | 0.076 |
| TN | *p* | 0.119 | 0.192 | 0.09 | 0.087 | 0.025 | 0.023 | -0.029 |
| | $R^2$ | 0.239 | 0.055 | 0.375 | 0.387 | 0.804 | 0.819 | 0.776 |
| TC | *p* | 0.159 | 0.176 | 0.171 | 0.156 | 0.088 | -0.003 | 0 |
| | $R^2$ | 0.113 | 0.08 | 0.089 | 0.121 | 0.384 | 0.979 | 0.996 |
| C:N | *p* | -0.013 | -0.087 | 0.03 | 0.022 | -0.007 | 0.022 | -0.017 |
| | $R^2$ | 0.896 | 0.392 | 0.766 | 0.826 | 0.945 | 0.83 | 0.867 |

*. Correlation is significant at the 0.05 level (2-tailed).

**. Correlation is significant at the 0.01 level (2-tailed).

N=100

Ammonia oxidation: AO; Heterotrophic Denitrification: HD

Supplementary Table S4 Details of the RT-qPCR analysis.

| Specificity | Primers | Sequence (5'-3') | Thermal profiles | Eff (%) | $R^2$ | Ref. |
|---|---|---|---|---|---|---|
| AOA *amo*A | Arch-amoAF | STAATGGTCTGGCTTAGACG | 95°C for 5 min; 95°C for 30 s, 55°C for 30 s, 72°C for 40 s, 40 cycles | 95.3 | 0.99 | 36 |
| | Arch-amoAR | GCGGCCATCCATCTGTATGT | | | | |
| AOB *amo*A | amoA-1F | GGGGTTTCTACTGGTGGT | 95°C for 5 min; 95°C for 30 s, 57°C for 30 s, 72°C for 40 s, 40 cycles | 86.8 | 0.99 | 37 |
| | amoA-2R | CCCTCKGSAAAGCCTTCTTC | | | | |
| *nir*K | nirK-876F | ATYGGCGGVCAYGGCGA | 95°C for 5 min; 95°C for 30 s, 55°C for 30 s, 72°C for 40 s, 40 cycles | 122.5 | 0.99 | 38 |
| | nirK-1040R | GCCTCGATCAGRTTRTGGTT | | | | |
| *nir*S | nirS-F | AACGYSAAGGARACSGG | 95°C for 5 min; 95°C for 30 s, 52.5°C for 30 s, 72°C for 30 s, 40 cycles | 97.3 | 0.99 | 38 |
| | nirs-R | GASTTCGGRTGSGTCTTSAYGAA | | | | |
| *nor*B | norB-F | GACAARHWVTAYTGGTGGT | 95°C for 5 min; 95°C for 30 s, 57.5°C for 30 s, 72°C for 40 s, 40 cycles | 91.5 | 0.98 | 39 |
| | norB-R | TGCAKSARRCCCCABACBCC | | | | |
| *nos*Z | nosZ-F | CGCRACGGCAASAAGGTSMSSGT | 95°C for 5 min; 95°C for 30 s, 54°C for 30 s, 72°C for 40 s, 40 cycles | 121.7 | 0.97 | 38 |
| | nosZ-R | CAKRTGCAKSGCRTGGCAGAA | | | | |

S:G/C; K: G/T; Y: C/T; V: G/A/C; R: A/G; H:; W: A/T; B: G/T/C

## Supplementary References


1. Uritskiy, G. V., DiRuggiero, J. & Taylor, J. MetaWRAP-A flexible pipeline for genome-resolved metagenomic data analysis. *Microbiome* **6**, 1–103 (2018).
2. Li, D., Liu, C., Luo, R., Sadakane, K. & Lam, T. MEGAHIT: An Ultra-fast single-node solution for large and complex metagenomics assembly via succinct de bruijn graph. *Bioinformatics* **31**, 1674–1676 (2015).
3. Langmead, B., Salzberg, S. Fast gapped-read alignment with Bowtie 2. *Nat. Methods* **9**, 357–359 (2012).
4. Li, H., Handsaker, B., Wysoker, A. A., Fennell, T., Ruan, J., Homer, N., Genome Project Data, P. The Sequence Alignment/Map format and SAM tools. *Bioinformatics* **25**, 2078–2079 (2009).
5. Alneberg, J., Bjarnason, B. S., de Bruijn, I., Schirmer, M., Quick, J., Ijaz, U. Z., Quince, C. Binning metagenomic contigs by coverage and composition. *Nat. Methods* **11**, 1144–1146 (2014).
6. Wu, Y., Simmons, B. A. & Singer, S. W. MaxBin 2.0: An Automated binning algorithm to recover genomes from multiple metagenomic datasets. *Bioinformatics* **32**, 605–607 (2016).
7. Kang, D. D., Li, F., Kirton, E., Thomas, A., Egan, R., An, H., & Wang, Z. MetaBAT 2: an adaptive binning algorithm for robust and efficient genome reconstruction from metagenome assemblies. *Peer J.* **7**, 7359 (2019).
8. Parks D. H., Chuvochina, M., Waite, D. W., Rinke, C., Skarshewski, A., Chaumeil, P. A., & Hugenholtz, P. Recovery of nearly 8,000 metagenome-assembled genomes substantially expands the tree of life. *Nat. Microbiol.* **2**, 1533–1542 (2017).
9. Parks, D. H., Imelfort, M., Skennerton, C. T., Hugenholtz, P. & Tyson, G. W. CheckM: Assessing the quality of microbial genomes recovered from isolates, single cells, and metagenomes.

*Genome Res.* **25**, 1043–1055 (2015).

10. Olm, M. R., Brown, C. T., Brooks, B., & Banfield, J. F. Drep: a tool for fast and accurate genomic comparisons that enables improved genome recovery from metagenomes through de-replication. *ISME J.* **11**, 2864–2868 (2017).
11. Uritskiy, G. V., DiRuggiero, J., & Taylor, J. MetaWRAP—a flexible pipeline for genome-resolved metagenomic data analysis. *Microbiome* **6**, 158 (2018).
12. Parks, D. H., Chuvochina, M., Waite, D. W., Rinke, C., Skarshewski, A., Chaumeil, P. A., & Hugenholtz, P. A standardized bacterial taxonomy based on genome phylogeny substantially revises the tree of life. *Nat. Biotechnol.* **36**, 996–1004 (2018).
13. Tu, Q., Lin, L., Cheng, L., Deng, Y. & He, Z. NCycDB: a curated integrative database for fast and accurate metagenomic profiling of nitrogen cycling genes. *Bioinformatics* **35**, 1040–1048 (2019).
14. Braker, G., Fesefeldt, A., & Witzel, K. P. Development of PCR primer systems for amplification of nitrite reductase genes (*nir*K and *nir*S) to detect denitrifying bacteria in environmental samples. *Appl. Environ. Microbiol.* **64**, 3769–3775 (1998).
15. Braker, G., Zhou, J., Wu, L., Devol, A. H., & Tiedje, J. M. Nitrite reductase genes (*nir*K and *nir*S) as functional markers to investigate diversity of denitrifying bacteria in pacific northwest marine sediment communities. *Appl. Environ. Microbiol.* **66**, 2096–2104 (2000).
16. Hall, B. G. Building Phylogenetic Trees from Molecular Data with MEGA. *Mol. Biol. Evol.* **30**, 1229–1235 (2013).
17. Adhikari, A., Nandi, S., Bhattacharya, I., Roy, M. D., Mandal, T., & Dutta, S. Phylogenetic analysis based evolutionary study of 16S rRNA in known *Pseudomonas sp. Bioinformation* **11(10)**, 474–480 (2015).

18. Wemheuer, F., Taylor, J.A., Daniel, R., Johnston, E., Meinicke, P., Thomas, T., & Wemheuer, B. Tax4Fun2: prediction of habitat-specific functional profiles and functional redundancy based on 16S rRNA gene sequences. *Environ. Microbiome* **15**, 11 (2020).
19. Qin, Y., Wang, S. Y., Wang, X. M., Liu, C. L., & Zhu, G. B. Contribution of ammonium-induced nitrifier denitrification to $N_2O$ in paddy fields. *Environ. Sci. Technol.* **57(7),** 2970–2980 (2023).
20. Daims, H., Lebedeva, E. V., Pjevac, P., Han, P., Herbold, C., Albertsen, M., Jehmlich, N., Palatinszky, M., Vierheilig, J., Bulaev, A., Kirkegaard, R. H., von Bergen, M., Rattei, T., Bendinger, B., Nielsen, P. H., & Wagner, M. Complete nitrification by *Nitrospira* bacteria. *Nature* **528(7583)**, 504–509 (2015).
21. Picone, N., Pol, A., Mesman, R., van Kessel, M. A. H. J., Cremers, G., van Gelder, A. H., van Alen, T. A., Jetten, M. S. M., Lücker, S., & Op den Camp, H. J. M. Ammonia oxidation at pH 2.5 by a new gammaproteobacterial ammonia-oxidizing bacterium. *ISME J.* **15**, 1150–1164 (2021).
22. Wang, D., Chen, Z., Sun, W., Hu, B. & Xu, S. Methane and nitrous oxide concentration and emission flux of Yangtze Delta plain river net. *Sci. China Ser. B: Chem*. **52**, 652–661 (2009).
23. Yang, L., Yan, W., Ma, P. & Wang, J. Seasonal and diurnal variations in $N_2O$ concentrations and fluxes from three eutrophic rivers in Southeast China. *J. Geogr. Sci.* **21**, 820 (2011).
24. Wang, J., Chen, N., Yan, W., Wang, B. & Yang, L. Effect of dissolved oxygen and nitrogen on emission of $N_2O$ from rivers in China. *Atmos. Environ.* **103**, 347–356 (2015).
25. Li, Q., Wang, F., Yu, Q., Yan, W., Li, X. & Lv, S. Dominance of nitrous oxide production by nitrification and denitrification in the shallow Chaohu Lake, Eastern China: Insight from isotopic characteristics of dissolved nitrous oxide. *Environ. Pollut*. **255**, 113212 (2019).

26. Wang, H., Zhang, L., Yao, X., Xue, B. & Yan W. Dissolved nitrous oxide and emission relating to denitrification across the Poyang Lake aquatic continuum. *J. Environ. Sci*. **52**, 130–140 (2017).

27. Zhou, Y., Xu, X., Han, R., Li, L., Feng, Y., Yeerken, S., Song, K., & Wang, Q. Suspended particles potentially enhance nitrous oxide ($N_2O$) emissions in the oxic estuarine waters of eutrophic lakes: Field and experimental evidence. *Environ. Pollut.* **252**, 1225–1234 (2019).

28. Gao, D., Hou, L., Li, X., Liu, M., Zheng, Y., Yin, G., Yang, Y., Liu, C., & Han, P. Exotic Spartina alterniflora invasion alters soil nitrous oxide emission dynamics in a coastal wetland of China. *Plant Soil* **442**, 233–246 (2019).

29. Gao, D., Hou, L., Liu, M., Li, X., Zheng, Y., Yin, G., Wu, D., Yang, Y., Han, P., Liang, X., & Dong, H. Mechanisms responsible for $N_2O$ emissions from intertidal soils of the Yangtze Estuary. *Sci. Total Environ*. **716**, 137073 (2020).

30. Musenze, R. S., Werner, U., Grinham, A., Udy, J., & Yuan, Z. G. Methane and nitrous oxide emissions from a subtropical estuary (the Brisbane River estuary, Australia). *Sci. Total Environ*. **472**, 719–729 (2014).

31. Hernandez, M.E. & Mitsch, W. J. Influence of hydrologic pulses, flooding frequency, and vegetation on nitrous oxide emissions from created riparian marshes. *Wetlands* **26**, 862–877 (2006).

32. Sun, Q. Q., Whitham, C., Shi, K., Yu, G. H., & Sun, X. W. Nitrous oxide emissions from a waterbody in the Nenjiang basin, China. *Hydrol. Res*. **43**, 862–869 (2012).

33. Li, X. F., Sardans, J., Qi, M. T., Ni, X. Y., Zhang, M. F., Penuelas, J., Yue, K., & Wu, F. Z. Nitrous oxide concentration and flux in Min River Basin of southeast China: Effects of land

use, stream order and water variables. *J. Hydrol*. **614** (2023).

34. Audet, J., Wallin, M.B., Kyllmar, K., Andersson, S., & Bishop, K. Nitrous oxide emissions from streams in a Swedish agricultural catchment. *Agr. Ecosyst. Environ.* **236**, 295–303 (2017).

35. Yang, L., Li, H. P., Wang, J. Spatial and Temporal Variability of Nitrous Oxide Emissions from a Large Subtropical Reservoir in Eastern China. *Pol. J. Environ. Stud*. **28**, 3497–3503 (2019).

36. Rotthauwe, J. H., Witzel, K. P., Liesack, W. The ammonia monooxygenase structural gene amoA as a functional marker: Molecular fine-scale analysis of natural ammonia-oxidizing populations. *Appl. Environ. Microbiol.* **63(12)**, 4704–4712 (1997).

37. Francis, C. A., Roberts, K. J., Beman, J. M., Santoro, A. E., Oakley, B. B. Ubiquity and diversity of ammonia-oxidizing archaea in water columns and sediments of the ocean. *Proc. Natl. Acad. Sci. U. S. A.* **102(41)**, 14683–14688 (2005).

38. Hallin, S., Jones, C. M., Schloter, M., Philippot, L. Relationship between N-cycling communities and ecosystem functioning in a 50-year-old fertilization experiment. *ISME J.* **3(5)**, 597–605 (2009).

39. Braker, G. & Tiedje, J. M. Nitric oxide reductase (*nor*B) genes from pure cultures and environmental samples. *Appl. Environ. Microbiol.* **69(6)**, 3476–83 (2003).
