# Supplementary material for: Ammonium-derived nitrous oxide is a global source in streams
Source: Nat Commun. 2024 May 14;15:4085. doi: 10.1038/s41467-024-48343-9 (PMC11094135; doi:10.1038/s41467-024-48343-9)
Supplement: Supplementary file 3 — Description of Additional Supplementary Files [file 41467_2024_48343_MOESM3_ESM.pdf]

Description of Additional Supplementary Files

File Name: Supplementary Data 1
Description: Details of the sampling sites and analyses on Site, Regional, and Global scale studies.

File Name: Supplementary Data 2
Description: Raw data of the physio-chemical characteristics and N2O-production rates detected by C_2H_2-inhibitor method.

File Name: Supplementary Data 3
Description: Raw data of ^{15}N-tracing *semi-in-situ* incubation and the contributions of N_2O-prodution pathways. a) Contribution and Rate of N_2O-prodution pathways; b) Raw data of 15N-tracing semi-in-situ incubation; c) Raw data of N_2O concentration in the incubation tubes.

File Name: Supplementary Data 4
Description: The contributions of N_2O-prodution pathways and Raw data of ^{15}N-^{18}O dual-isotope incubation. a) Contributions of N_2O-prodution pathways; b) Raw data of N_2O reference; c) Raw data of N_2O samples; d) Raw data of the chemical-transformed N_2.

File Name: Supplementary Data 5
Description: Processing information and NCBI sequence accession number of Metagenomic raw data.

File Name: Supplementary Data 6
Description: Information and NCBI sequence accession number of Metagenome-assembled genomes (MAGs).
