# Supplementary material for: Ammonium-derived nitrous oxide is a global source in streams
Source: Nat Commun. 2024 May 14;15:4085. doi: 10.1038/s41467-024-48343-9 (PMC11094135; doi:10.1038/s41467-024-48343-9)
Supplement: Supplementary file 10 — Reporting Summary [file 41467_2024_48343_MOESM10_ESM.pdf]

Corresponding author(s): Guibing Zhu

Last updated by author(s): Apr 25, 2024

Reporting Summary

Nature Portfolio wishes to improve the reproducibility of the work that we publish. This form provides structure for consistency and transparency in reporting. For further information on Nature Portfolio policies, see our Editorial Policies and the Editorial Policy Checklist.

Statistics

For all statistical analyses, confirm that the following items are present in the figure legend, table legend, main text, or Methods section.

n/a	Confirmed	
☐	☒	The exact sample size (*n*) for each experimental group/condition, given as a discrete number and unit of measurement
☐	☒	A statement on whether measurements were taken from distinct samples or whether the same sample was measured repeatedly
☐	☒	The statistical test(s) used AND whether they are one- or two-sided<br>*Only common tests should be described solely by name; describe more complex techniques in the Methods section.*
☐	☒	A description of all covariates tested
☒	☐	A description of any assumptions or corrections, such as tests of normality and adjustment for multiple comparisons
☐	☒	A full description of the statistical parameters including central tendency (e.g. means) or other basic estimates (e.g. regression coefficient) AND variation (e.g. standard deviation) or associated estimates of uncertainty (e.g. confidence intervals)
☐	☒	For null hypothesis testing, the test statistic (e.g. *F*, *t*, *r*) with confidence intervals, effect sizes, degrees of freedom and *P* value noted<br>*Give P values as exact values whenever suitable.*
☒	☐	For Bayesian analysis, information on the choice of priors and Markov chain Monte Carlo settings
☒	☐	For hierarchical and complex designs, identification of the appropriate level for tests and full reporting of outcomes
☐	☒	Estimates of effect sizes (e.g. Cohen's *d*, Pearson's *r*), indicating how they were calculated

Our web collection on statistics for biologists contains articles on many of the points above.

Software and code

Policy information about availability of computer code

Data collection	No software was used for data collection
Data analysis	Data analysis softwares used in this study included Kneaddata (github.com/biobakery/kneaddata), FastQC (Babraham Bioinformatics, Babraham institute, Cambridge, UK), Megahit, Bowtie2, Samtools, MetaWRAP version 1.2.1, CheckM, dRep, MetaWRAP (salmon algorithm), GTDB-Tk v2.3.0, KEGG, NCyc, COG, and GO databases, MEGA 11, Statistical Product and Service Solutions 18.0 software.

For manuscripts utilizing custom algorithms or software that are central to the research but not yet described in published literature, software must be made available to editors and reviewers. We strongly encourage code deposition in a community repository (e.g. GitHub). See the Nature Portfolio guidelines for submitting code & software for further information.

Data

Policy information about availability of data

All manuscripts must include a data availability statement. This statement should provide the following information, where applicable:

- Accession codes, unique identifiers, or web links for publicly available datasets
- A description of any restrictions on data availability
- For clinical datasets or third party data, please ensure that the statement adheres to our policy

The data generated in this study are provided within the article, Supplementary Information, and Supplementary Data files. Metagenomic sequencing data and

metagenome-assembled genomes are available in the NCBI Sequence Read Archive (SRA) under the accession codes PRJNA943572 and PRJNA1031250, respectively.

Research involving human participants, their data, or biological material

Policy information about studies with human participants or human data. See also policy information about sex, gender (identity/presentation), and sexual orientation and race, ethnicity and racism.

Reporting on sex and gender	This information has not been collected.
Reporting on race, ethnicity, or other socially relevant groupings	This information has not been collected.
Population characteristics	This information has not been collected.
Recruitment	This information has not been collected.
Ethics oversight	This information has not been collected.

Note that full information on the approval of the study protocol must also be provided in the manuscript.

Field-specific reporting

Please select the one below that is the best fit for your research. If you are not sure, read the appropriate sections before making your selection.

☐ Life sciences ☐ Behavioural & social sciences ☒ Ecological, evolutionary & environmental sciences

For a reference copy of the document with all sections, see nature.com/documents/nr-reporting-summary-flat.pdf

Ecological, evolutionary & environmental sciences study design

All studies must disclose on these points even when the disclosure is negative.

Study description

An increasing number of studies indicated that global riverine N2O emissions have increased over 4-fold in the Anthropocene. It appears that especially the hyporheic zones in small streams may contribute about 85% of this nitrous oxide emission. However, the mechanisms and pathways controlling hyporheic N2O production in stream ecosystems remain unknown and poorly modeled.
In this context, the aim of this study was to investigate which microbial sources and mechanisms are responsible for hyporheic N2O production in open water and riparian zone sediments. We first investigated the spatiotemporal characteristics along transects across the Baiyangdian riverine networks, the largest riverine network in the North China Plain, using the isotopic 15N-18O & 15N tracing technology, quantitative reverse transcription PCR (RT-qPCR), and metagenome analysis at site and regional scales. The North China Plain covers 300,000 km2 and accounts for 23% of the Chinese cropland area. However, it accounts for more than 25% of the global fertilizer N used, making it a global N2O emission hotspot. In spite of this, only few papers have investigated the N2O emissions from streams in China in general and the North China Plain in particular. After this, we compared these results to those measured globally in temperate and tropical streams so as to obtaining a comprehensive conclusion.

For the RT-qPCR analysis, RNA was extracted from three parallel site-scale sediment cores from both the riparian zone and riverbed sediments using a RNeasy Power Microbiome RNA Isolation Kit (QIAGEN, Hilden, Germany) according to the manufacturer's protocol. RNA quality and concentration were estimated using a NanoDrop 2000 Spectrophotometer (NanoDrop Technologies, Wilmington, DE, USA). The three biological biologically RNA samples were used to carried out the RT-qPCR analysis. All RT–qPCR analyses were performed on a sequence detection system (ABI 7500; Applied Biosystems, Foster City, CA, USA) with SYBR-Green fluorescent dye (TaKaRa). The copy numbers of N2O-related genes were quantified by using the specific primers for the archaeal amoA gene with Arch-amoAF and Arch-amoAR, the bacterial amoA gene with amoA-1F and amoA-2R, the denitrifier and ammonia oxidizer nirK gene with nirK-876F and nirK-1040R, the nirS gene with nirS-F and nirS-R, the denitrifier and ammonia oxidizer norB gene with norB-F and norB-R, and the nosZ gene with nosZ-F and nosZ-R. all tests were conducted in triplicate with amplification efficiencies between 90% and 110% and correlation coefficients (R2) above 0.98.

Research sample

Riverine N2O emissions have increased 4-fold since 1900 and are important components of the global N2O budget. A 2020 global modeling study showed that small streams (lower-order streams rather than high-order streams) contribute up to 85% of the global riverine nitrous oxide (N2O) emissions, most of which are produced in hyporheic zones (beneath stream beds where stream waters exchange with adjacent sediments). However, the mechanisms and pathways contributing to N2O production in agricultural soils have not yet been well investigated.
Our research extends from site scale to regional scale and then to global scale. The Baiyangdian riverine network (113°40′~116°20′E, 38°10′~40°10′N) in the Haihe River Basin, with a total area of 3.12×104 km2, is the largest riverine network in the North China Plains. The river network density is 0.50-0.99 km/km2, representing the upper level in China, with significant temporal distribution differences. Its upper and middle regions receive 30-40 first- or second-order tributaries or small streams with a total length of approximately 3000 kilometers. These small streams drain various land areas in agricultural, urban, and mountain regions and are affected by various human activities and by agricultural fertilization practices. It is a good study area for our topic. At the site scale, we collected three parallel sediment cores at the riparian zone and open water zone, respectively (Figure 1). At the regional scale, 25 sampling sites (5 rivers * 5 sampling sites) in the Baiyangdian riverine network were sampled in riparian zone and open water sediments, respectively, in both rainy and dry seasons. Therefore, metagenomic analysis was performed at 100 sample sites (5 rivers

	* 5 sampling sites * 2 zones * 2 seasons) (Figure 2). Based on site and regional scale results, the global-scale study covered 28 low-order agricultural streams, regardless of seasons and zones (Figure 4). All the above sampling sites were collected in triplicate.
Sampling strategy	First, a site-scale study was conducted on one of these small streams, the Xiaoqinghe River. Nine parallel in situ sediment cores (0–20 cm depth) were collected separately from the riparian (2 m away from the water–soil transition zone) and riverbed (the middle of the river) zones. Six sediment cores were subjected to semi-in situ 15N tracer assays, and the other three were subjected to 0.01% C2H2 inhibitor assays and molecular analysis. Furthermore, a regional-scale study was conducted on five rivers in the Baiyangdian riverine network. A total of 100 sediment samples were collected in the riparian zone and from riverbed sediments in the five rivers at five sampling sites along each river during the dry (Jan to Mar 2021) and rainy (Aug to Oct 2021) seasons. Then, we chose steams located adjacent to farmlands as the study sites, characterized by their land use type being cropland land45. Twenty-eight low-order agricultural streams were sampled in major river basins worldwide, including the Mississippi River, Colorado River (North America), Amazon River, Ucayali River (South America), Elbe River, Weser River, Po River (Europe), Niger River, Zambezi River (Africa), Murray River (Oceania), Indus River, Yangtze River, Yellow River, Pearl River, Yarlung Zangbo River, Huai River, Liao River, Songhuajiang River, and Heilongjiang River (Asia) .
Data collection	We collected stream/river sediments around the world between 2013 to 2021 through our team and the assistance of collaborators, whose names has been detailed and acknowledged in the Supplementary Data S1. All the sediments were placed in individual sterile plastic bags, stored on ice immediately, and then transported to our laboratory at a cooler as soon as possible for subsequent analyses. In our laboratory, the samples were analyzed by the co-authors Shanyun Wang, Bangrui Lan, Longbin Yu, Manyi Xiao, Liping Jiang, Yu Qin, Yucheng Jin, Yuting Zhou, Gawhar Armanbek, Jingchen Ma, and Manting Wang.
Timing and spatial scale	We collected stream/river sediments around the world through our team and the assistance of collaborators between 2013 to 2021. Among them, the regional scale samples were collected from the riparian zone and riverbed sediments at five sampling sites in five rivers along each river during the dry (Jan to Mar 2021) and rainy (Aug to Oct 2021) seasons. Details were showed in the Supplementary Data S1.
Data exclusions	For metagenomic library sequencing, raw metagenomic reads with low-quality nucleotides and reads with any ambiguous base calls were excluced. The MAGs with a completion degree lower than 75% and a pollution degree of higher than 15% were excluded.
Reproducibility	All study sites were sampled in triplicate. Meanwhile, all the N2O production rates/sources, transcript abundances of N2O-related functional genes and the physical and chemical properties were analyzed in triplicate in our laboratory. All attempts to repeat the experiment were successful.
Randomization	Allocation was random. For N2O production rate/source analysis, 5 g of homogeneous fresh sediment with 25 ml overlying water were placed into at least 12 parallel 60-mL glass serum vials (Ochs Laborbedarf, Germany). Four treatments enriched in 18O and 15N were completely applied at random vails in triplicate. This approach is designed to minimize incubation differences.
Blinding	The data in our study predominantly comes from experiments with the unknown environmental samples collected from the definite study locations. Thus, single-blinding was relevant to this study.

Did the study involve field work? ☒ Yes ☐ No

Field work, collection and transport

Field conditions	The information of ambient temperatures have been detailed in the Supplementary Data S1.
Location	The information of elevation and geographical coordinates have been detailed in the Supplementary Data S1.
Access & import/export	The collections of sediments in this study did not involve sensitive or prohibited areas.
Disturbance	We collected the appropriate amount of field samples and transported them back to the laboratory for analysis, with almost no impact on the field environment.

Reporting for specific materials, systems and methods

We require information from authors about some types of materials, experimental systems and methods used in many studies. Here, indicate whether each material, system or method listed is relevant to your study. If you are not sure if a list item applies to your research, read the appropriate section before selecting a response.

Materials & experimental systems

n/a	Involved in the study	
☒	☐	Antibodies
☒	☐	Eukaryotic cell lines
☒	☐	Palaeontology and archaeology
☒	☐	Animals and other organisms
☒	☐	Clinical data
☒	☐	Dual use research of concern
☒	☐	Plants

Methods

n/a	Involved in the study	
☒	☐	ChIP-seq
☒	☐	Flow cytometry
☒	☐	MRI-based neuroimaging

Plants

Seed stocks	n/a
Novel plant genotypes	n/a
Authentication	n/a
